# Brain-Derived Neurotrophic Factor and Its Potential Therapeutic Role in Stroke Comorbidities

**DOI:** 10.1155/2020/1969482

**Published:** 2020-01-27

**Authors:** Wei Liu, Xiaohui Wang, Margaret O'Connor, Guan Wang, Fang Han

**Affiliations:** ^1^Department of Neurology, Beijing Haidian Hospital, Beijing, China; ^2^Department of General Surgery, Xuanwu Hospital, Capital Medical University, Beijing, China; ^3^Department of Biology, Boston University, Boston, MA, USA; ^4^School of Pharmaceutical Sciences, Tsinghua University, Beijing, China

## Abstract

With the rise in the aging global population, stroke comorbidities have become a serious health threat and a tremendous economic burden on human society. Current therapeutic strategies mainly focus on protecting neurons from cytotoxic damage at the acute phase upon stroke onset, which not only is a difficult way to ameliorate stroke symptoms but also presents a challenge for the patients to receive effective treatment in time. The brain-derived neurotrophic factor (BDNF) is the most abundant neurotrophin in the adult brain, which possesses a remarkable capability to repair brain damage. Recent promising preclinical outcomes have made BDNF a popular late-stage target in the development of novel stroke treatments. In this review, we aim to summarize the latest progress in the understanding of the cellular/molecular mechanisms underlying stroke pathogenesis, current strategies and difficulties in drug development, the mechanism of BDNF action in poststroke neurorehabilitation and neuroplasticity, and recent updates in novel therapeutic methods.

## 1. Introduction

Stroke is a destructive cerebrovascular disease that occurs when a blood vessel carrying oxygen and nutrients to the brain either bursts or is blocked by a clot. As the second leading cause of human death and the third leading cause of disability, stroke claims around 6.5 million lives and 44 million disability-adjusted life-years (DALYs) globally every year. Thus, this disease has become an enormous threat to human health and a huge burden to the healthcare system worldwide [1–4]. So far, the only FDA-approved medication for ischemic stroke is the tissue plasminogen activator (tPA) when applied within 3 hours of an acute ischemic stroke attack, which therefore benefits only a small portion of the patients (2-5%) [5–7]. A great deal of effort has been made toward developing neuroprotectants, which mostly aim to block individual cytotoxic pathways in the early stages of stroke pathogenesis. However, clinical trials for these neuroprotective drugs have had little success, possibly due to the involvement of complex mechanisms in the cytotoxic and neuronal death processes during stroke [8, 9]. This failure demanded a change in strategy for the development of stroke therapeutics.

In recent years, neurorehabilitation and recovery have become new popular directions in the scientific research and drug development of stroke. As a result, neurotrophins have become a rising star in this field. Of particular interest is BDNF, due to its high cerebral abundance and ability to attenuate neuronal injury and repair brain damage. Preclinical studies using BDNF, or its mimetics, have generated promising results in the treatment of acute brain injuries and are on track for use in clinical trials in the near future [10–12]. In this review, we aim to summarize recent progress in the research and development of stroke therapeutics, including the challenges and potential of BDNF and its downstream signaling pathways as new targets.

## 2. Stroke Pathogenesis: The Molecular Mechanisms

By continuing to expand our knowledge of the molecular mechanisms underlying the pathogenesis of stroke, we stand a better chance in the fight against this devastating disease. Thanks to decades of joint effort across human society, we now understand more about what occurs at the molecular level in a poststroke brain, which has conversely helped scientists to study this disease in more detail as well as to aid them in developing new therapeutic plans for treatment.

**Figure 1:**
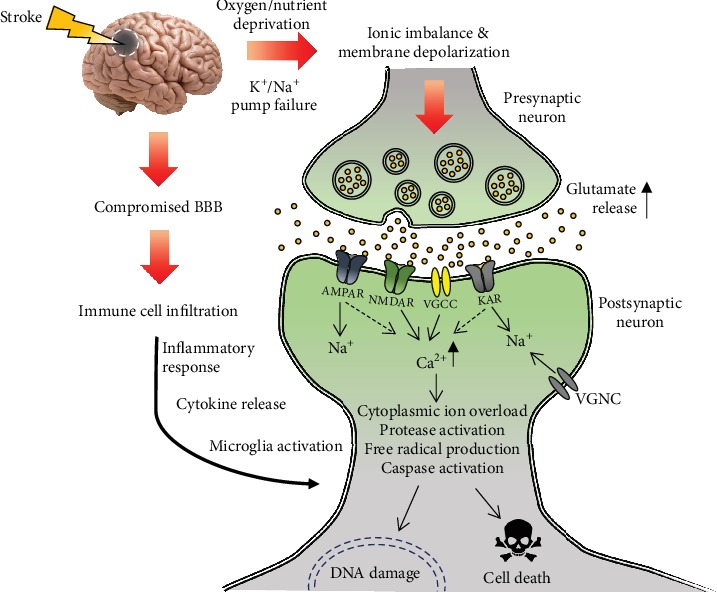
A schematic diagram depicting the pathogenesis of stroke in neurons. Upon the onset of stroke, reduced oxygen and nutrient supplies rapidly lead to the failure of ATP-dependent Na^+^/K^+^ pumps causing ionic imbalance and cell membrane depolarization, resulting in presynaptic overrelease of neurotransmitters including glutamate into the synaptic cleft. Activation of postsynaptic glutamate receptors AMPAR, NMDAR, and KAR leads to large volume Na^+^ and Ca^2+^ influxes, further cell membrane depolarization of the postsynaptic neuron, and opening of the membrane potential-sensitive VGNCs and VGCCs. This allows even more Na^+^ and Ca^2+^ to flow into the cell which causes cytoplasmic ion overload, protease activation, production of free radicals, caspase activation, and eventually DNA damage and neuronal cell death. Meanwhile, as the BBB is compromised during stroke, immune cells from the blood start to infiltrate the brain to elicit inflammatory responses, such as cytokine release and microglial cell activation, which further exacerbate the brain damage and injury. BBB: blood-brain barrier; AMPAR: *α*-amino-3-hydroxy-5-methyl-4-isoxazolepropionic acid receptor; NMDAR: *N*-methyl-D-aspartic acid receptor; KAR: kainic acid receptor; VGCC: voltage-gated calcium channel; VGNC: voltage-gated sodium channel.

Although stroke is classified as ischemic or hemorrhagic depending on the pathophysiology (ischemia or hemorrhage), the clinical presentation of stroke in patients is largely the same independent of the cause [13]. In the case of ischemia, clotting within the brain cuts off the core supply of oxygen and glucose causing a drastic reduction in the peri-infarct area. Influenced cells in the ischemic area experience energy depletion or reduction, leading to failures in ATP-dependent pumps and ionic imbalance. This subsequently results in cell membrane depolarization and increased permeability, accompanied with the release of excitotoxic neurotransmitters and activation of glutamate receptors. Through the opened glutamate receptor ion channels, Ca^2+^ and Na^+^ influxes cause an overload in cytoplasmic ions, which subsequently activate lipid peroxidases, proteases, and phospholipases. High levels of Ca^2+^, Na^+^, and ADP result in the production of oxygen radicals and the opening of mitochondrial permeability transition pores, which eventually triggers apoptosis cascades. Thereafter, immune responses follow with microglia activation, proinflammatory cytokine release, and immune cell infiltration through the compromised blood-brain barrier (BBB) (Figure 1) [14].

In the case of intracerebral hemorrhage, in addition to the pathological processes triggered by the loss of oxygen and glucose supply, burst blood vessels add complexity to the disease by introducing even more components that are deleterious to the brain. These components include nucleic acids, proteins, and lipids as well as components of the complement system, immunoglobulin, and cells that release toxic prooxidative and proinflammation substances. Moreover, continuous secondary damage is caused by hemoglobin released by lysed red blood cells. It degrades into heme and iron, which further generate free radicals that damage macromolecules in healthy cells [15]. In this kind of stroke, the clean-up of the hematoma is important for long-term recovery after hemorrhagic stroke.

## 3. Current Barriers and Strategies to Treat Stroke

Due to the different physiological conditions of stroke-insulted brains, along with the variety of possible causes and subsequent pathological events, there are enormous complexities and barriers to the effective treatment of stroke. To date, the only FDA-approved drug for ischemic stroke is the tPA that breaks down blood clots in the brain. However, as shown in clinical studies, treatment with tPA is only effective if administered within 4.5 hours postischemia. Although it was recently reported that the timeframe for effective thrombolysis treatment extends up to 9 hours after the onset of a stroke, this time window remains narrow [5–7], not to mention the side effects of tPA, which include enhanced risk of brain hemorrhage. In fact, the number of patients actually benefiting from tPA is very limited (2-5%) [16].

Thus far, strategies to treat stroke have mainly focused on intervention in its pathological process. *N*-Methyl-D-aspartic acid receptor (NMDAR) antagonists, free radical scavengers, and molecules reducing immune cell infiltration have already shown promising results in animal models [8, 17–20]; however, all of these treatments have unfortunately failed in clinical trials [8, 9]. In general, the failures could be attributed to several challenges. Firstly, there is the narrow time window for treatment. Upon stroke, excitotoxic neurotransmitters, Ca^2+^, and free radicals start to accumulate rapidly and disturb normal cellular functions immediately. These therapies are not very practical for the timely treatment of patients before irreversible damage occurs. Second, complex underlying mechanisms make it difficult to target a single molecule or pathway to efficiently attenuate all deleterious effects. Third, the pathological mechanisms of stroke remain largely unclear. Fourth, current animal models cannot represent the full scope of stroke as seen in human patients, especially the disease's unpredictable location and severity.

Nevertheless, these previous failures provide us with valuable knowledge and experience to reconsider our current therapeutic strategies and to begin focusing on other potential plans. New strategies include targeting cell death amelioration (neuroprotection) and repairing and regeneration (neurorehabilitation and recovery) in longer therapeutic windows which may eventually meet our needs [14, 21, 22]. Although in recent years there have been many reports of beneficial effects from off-label rehabilitative drug prescriptions that claim to alleviate symptoms of stroke patients, there remains no available neuroprotective medication to mitigate brain damage in acute or later phases poststroke. It is vital that future efforts change their focus and strategy in order to develop effective drugs for the treatment of stroke.

## 4. Neurotrophins: Potential New Candidates for Stroke Rehabilitation

**Figure 2:**
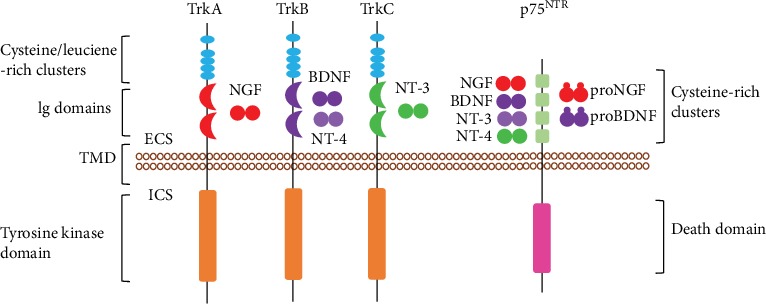
Schematic of neurotrophins and their target receptors. NGF, BDNF/NT-4, and NT-3 bind to TrkA, TrkB, and TrkC, respectively, with high affinity. While these neurotrophins can all also bind to p75^NTR^ with low affinity, the proneurotrophins proNGF and proBDNF can bind and activate the p75^NTR^ receptor with high affinity. Trk: tropomyosin receptor kinase; NGF: nerve growth factor; BDNF: brain-derived neurotrophic factor; NT: neurotrophin; Ig: immunoglobulin; TMD: transmembrane domain; ECS: extracellular space; ICS: intracellular space. This cartoon is adopted from Sanchez-Sanchez et al. with modifications [24].

Neurotrophins are a class of secreted proteins essential for the growth, differentiation, development, survival, and recovery of the nervous system [23]. Comprised of the nerve growth factor (NGF), BDNF, neurotrophin-3 (NT-3), and neurotrophin-4/5 (NT-4/5), neurotrophins bind to their high-affinity tyrosine kinase receptors (Trks), TrkA, TrkB, and TrkC, as well as their low-affinity receptor, p75^NTR^ (details are shown in Figure 2). Upon binding by neurotrophins, the Trks form homodimers and their intracellular receptor tyrosine kinase domains autophosphorylate each other to initiate downstream cascades of signaling transduction which lead to protein regulation and functional changes in the cell. Among the neurotrophins, BDNF is the most abundant in the adult brain, and extensive studies have reported on its neuroprotective effects in various neurological disorders, including both neurodegenerative diseases and acute brain injuries. This made BDNF a good candidate for the new direction of therapeutic strategies to treat stroke.

BDNF was the second neurotrophic factor to be identified when it was discovered by Barde et al. in 1982 [24], following the characterization of NGF by Nobel Laureates Levi-Montalcini and Cohen in 1956 [25, 26]. After three decades of extensive research and use in translational medicine, we now possess a wealth of knowledge regarding the biological properties and physiological functions of this protein.

BDNF plays a significant role in the development and functioning of the central nervous system. It is broadly involved in synapse maturation, synaptic plasticity, neurite outgrowth and arborization, and maintenance of normal cognitive function, while dysfunction of BDNF may contribute to the progression of multiple neurological diseases and psychiatric disorders [27–32]. The beneficial effects of BDNF in chronic neurological diseases and psychiatric disorders have been extensively reviewed in recent years [27, 33–35]. The prosurvival and neuroprotective functions of BDNF are mainly drawn from two signaling pathways activated by TrkB: the phosphatidylinositol 3-kinase (PI3K)/Akt and the mitogen-activated protein kinase/extracellular-signal-regulated kinase (MAPK/ERK) pathways. They both play significant roles in the cell cycle, division, and survival, by regulating the level and activity of certain transcription factors [35]. Therefore, BDNF appears to be a prime candidate for use as a stroke treatment.

## 5. Role of BDNF in Stroke and Poststroke Rehabilitation

BDNF plays a significant role in the prognosis, pathogenesis, and rehabilitation of stroke. It is well established that low levels of circulating BDNF are associated with a high risk of stroke and poor recovery, while BDNF expression in the brain is acutely stimulated by a stroke [36–40]. Clinically, positive outcomes have been demonstrated by using several stroke treatments that manipulate BDNF levels, including administration of hormones and neurotransmitter-targeting compounds, transplantation of stem cells, and regulation of other related genes [41–46]. We will now summarize the major, recent findings regarding stroke treatment and try to delineate the potential therapeutic capabilities of BDNF by focusing on different aspects of a stroke's cellular and molecular mechanisms.

### 5.1. BDNF Attenuates Stroke-Induced Cell Death

**Figure 3:**
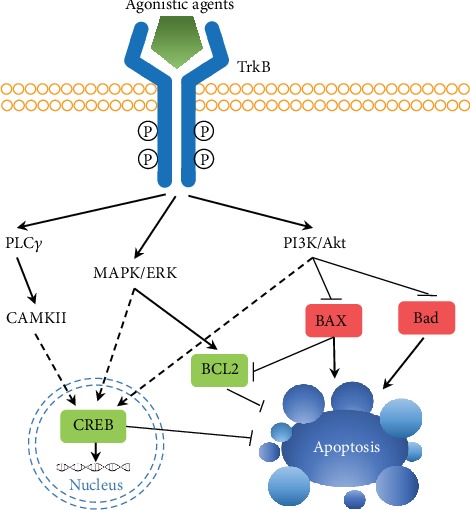
Activation of the TrkB receptor by specific agonists triggers downstream signaling cascades to induce transcription and suppress apoptosis. Upon binding to the TrkB receptor, specific agonistic agents induce the formation of a TrkB homodimer and autophosphorylation of the intracellular tyrosine kinase domains, as well as the activation of the PLC*γ* (phospholipase C gamma), MAPK/ERK, and PI3K/Akt signaling pathways. While the latter two pathways suppress apoptosis by interacting with apoptosis-regulating proteins, such as BCL2, BAX, and Bad, all three pathways activate CREB-mediated transcription of prosurvival genes and thus protect neurons from apoptosis.

As mentioned previously, through the activation of its downstream PI3K/Akt and MAPK/ERK pathways, BDNF is capable of protecting neurons from apoptosis, a critical event that directly leads to neuronal cell death and brain damage during stroke (Figure 3) [47–50]. The neuroprotective effects of both sex hormones and antioxidants to stroke may be exerted *via* these two pathways. In the rat model of middle cerebral artery occlusion (MCAO), poststroke administration of progesterone increased BDNF expression, attenuated apoptosis, and reduced neuronal injury *via* the PI3K/Akt pathway [51], while combinatorial treatment of progesterone and vitamin D protected neuronal cells from ischemia/reperfusion- (I/R-) induced apoptosis by increasing B-cell leukemia/lymphoma 2 (BCL-2) expression and suppressing caspase-3 cleavage *via* the BDNF-TrkB-ERK pathway (Figure 3) [52]. Antioxidants from natural products and from endogenous sources were also found to be capable of protecting neurons from I/R-induced apoptosis. A key enzyme catalyzing heme oxidative degradation and maintaining redox homeostasis in the cell, heme oxygenase-1 (HO-1), was upregulated by progesterone and vitamin D hormone during treatment of ischemic strokes [52]. Moreover, overexpression of HO-1 in the CA1 region of the hippocampus, prior to ischemia, significantly protected the brain from I/R injury. This led to increased relative ratio of BCL-2/BAX (BCL-2-associated X protein) and decreased cleaved-caspase-3 protein levels and, thus, less apoptosis. Similarly, when a natural isoflavonoid extracted from the root of *Pueraria*, puerarin, was administered 1 hour after I/R, there was a stimulation of BDNF expression in astrocytes and further suppression of apoptosis due to the promotion of BCL-2 and reduction in BAX expression [44].

In addition to apoptosis, a few novel types of cell death that play significant roles in the pathologic process of stroke have been identified, such as autophagy [53], necroptosis [10, 54], ferroptosis [55], parthanatos [56], and pyroptosis [57]. Although the role of BDNF in most of these cell deaths remains largely elusive, the latest evidence suggests that BDNF-TrkB signaling may protect neurons under ischemic insult from ferroptosis and necroptosis. Ishii et al. recently proposed that BDNF protects neurons from ferroptosis-like cell death *via* circadian activation of nuclear factor-E2-related factor 2 (Nrf2) in astrocytes [58]. Meanwhile, suppression of both neuronal cell apoptosis and necroptosis was observed by a new high-affinity TrkB agonistic antibody which thus reduced the infarct size and facilitated functional recovery in the rat MCAO model [10]. Experimental evidence from other disease models suggests this antinecroptotic effect may be mediated by the MAPK/ERK and PI3K/Akt pathways [59, 60]. Although more investigations are needed, these newly discovered neuronal cell death types may serve as novel targets for stroke treatment in the future (extensively reviewed by Fricker et al. [61]).

### 5.2. BDNF Promotes Neurite Outgrowth and Neurogenesis in Poststroke Rehabilitation

Neurite outgrowth and neurogenesis are hallmarks of the regenerative capability of the brain after injury, and the BDNF-TrkB signaling pathway plays a pivotal role in these processes [62–64]. In a cortical cell culture derived from endothelial nitric oxide synthase (eNOS) knockout mice, the reduced neurite outgrowth is shown to be caused by a BDNF-TrkB reduction [45]. Niacin, one of the most effective medications currently in clinical use for increasing high-density lipoprotein cholesterol, was tested in the rat MCAO model and found to promote neurite outgrowth and to increase synaptophysin expression, *via* activation of the BDNF-TrkB pathway [65].

Testosterone also exerts beneficial effects in stroke treatment by promoting neurogenesis and neurite outgrowth, which may be due to activation of the BDNF-TrkB pathway. Although testosterone was initially found to be deleterious in the acute phase of stroke, it was later suggested that testosterone treatment after ischemia could significantly enhance functional recovery in rats by increasing BDNF levels in both serum and the brain of experimental rats compared to untreated controls or those treated with testosterone and flutamide (an antiandrogen) [66].

Cilostazol is another clinically used medication found to have preventive effects on stroke [43, 67]. Cilostazol is a type 3 phosphodiesterase (PDE3) inhibitor that activates cAMP-responsive element binding protein (CREB) signaling and is commonly used to improve the symptoms of a certain blood flow problem in the legs (intermittent claudication). Post-I/R treatment with cilostazol was found to reduce infarct size, attenuate brain injury, and promote neurogenesis in a mouse MCAO model. These rehabilitative effects of cilostazol were most likely mediated by the activation of the BDNF-TrkB pathway, given that the number of BDNF-positive astrocytes in both the ipsilateral subventricular zone (SVZ) and the peri-infarct area was found to be significantly increased after treatment [43].

### 5.3. BDNF Facilitates Functional Recovery in Poststroke Rehabilitation

Functional recovery, the final outcome of poststroke rehabilitation, is a key endpoint for stroke patients in clinical trials, as well as for the experimental animals used in preclinical/scientific research. In recent years, novel therapeutic plans (e.g., gene/cell therapy) have greatly facilitated poststroke functional recovery. In an intracerebral hemorrhage mouse model, intracranial implantation of BDNF-overexpressing 3T3 fibroblasts significantly promoted poststroke neurogenesis and functional recovery [68]. In another study, intracerebral transplantation of genetically modified, BDNF-overexpressing human mesenchymal stem cells (MSCs) was achieved, resulting in significantly improved functional recovery in the rat MCAO model [69].

Erythropoietin (EPO) is a hematopoietic cytokine that shows neuroprotective effects in stroke by enhancing angiogenesis and neurogenesis and by upregulating synaptic plasticity-related genes, including BDNF [70]. Postischemia treatment with recombinant human EPO (rhEPO) in the rat MCAO model significantly increased the density of cerebral microvessels and the levels of the vascular endothelial growth factor (VEGF) and BDNF in the brain. Intriguingly, rhEPO was not able to induce BDNF expression in neurospheres from the SVZ, which implies the contribution of paracrine BDNF from endothelial cells in the neurogenesis and subsequent functional improvement [71].

In addition to administration of therapeutic agents and manipulation of gene expression, physical exercise was also capable of promoting BDNF expression and improving the neurological outcome after experimental stroke (reviewed by Alcantara et al. [72]). Ke et al. claimed that voluntary exercise is correlated with an increase in hippocampal BDNF levels and functional recovery [73]. In line with this, treadmill exercise increased only mature BDNF but not proBDNF in the cortex of rats that had experienced cortical ischemic stroke [74]. It was further reported that compared to those who were sedentary or who performed high-intensity exercise, MCAO rats who performed low-intensity exercise exhibited the greatest recovery in spatial memory and had a significant increase in PSD-95 and BDNF expression [75].

### 5.4. BDNF Induces Neuroplasticity in Poststroke Rehabilitation

The significant role that BDNF plays in the regulation and maintenance of synaptic plasticity has been extensively investigated and well described in recent decades. BDNF is well known for its involvement in Hebbian-type long-term potentiation (LTP) and long-term depression (LTD) and, more recently, in homeostatic synaptic plasticity (HSP) [30, 76]. However, unlike the synaptic plasticity that is crucial for normal cognitive function, the neuroplasticity discussed in poststroke rehabilitation mainly refers to the brain's capability to recover from damage in order to restore its normal structure and function.

The crucial role of BDNF in neuroplasticity was primarily revealed by studies that focused on the poststroke treatment of aphasia and lost motor function, which are largely mediated by the process of neuroplasticity [77, 78]. In previous studies using rat ischemia models, it was found that when BDNF synthesis was blocked, the beneficial effects on the recovery of skilled reaching were mostly negated [79], while intravenous administration of BDNF significantly enhanced the functional motor recovery of the treated rats, compared to the untreated controls [80, 81]. Behavioral and physical therapies, such as aerobic/physical exercise, transcranial direct current stimulation (tDCS), and extremely low-frequency electromagnetic field therapy (ELF-EMF), were all found to increase the blood and/or brain levels of BDNF [72, 77, 82–85]. In addition, BDNF-mediated learning memory may also be partially involved in the poststroke rehabilitation of motor function and language relearning [86–88].

## 6. BDNF as a Therapeutic Agent in the Treatment of Stroke

**Figure 4:**
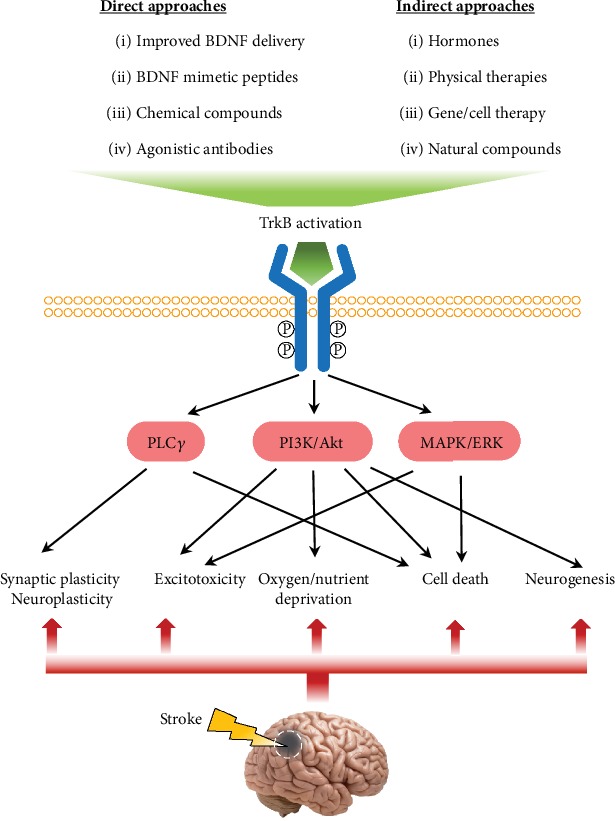
Summary of approaches to enhance TrkB activation and their effect on stroke treatment and recovery. Multiple strategies could be utilized to activate the TrkB receptor to serve in the treatment and recovery of stroke comorbidities. Direct approaches include the modification and improvement of BDNF for better delivery and the development of BDNF mimetic peptides, small molecule compounds, and specific agonistic antibodies. Indirect approaches include hormones, physical therapies, and natural compounds that can stimulate the endogenous expression of BDNF and gene/cell therapy to overexpress BDNF. TrkB receptor activation directly triggers and stimulates downstream signaling cascades, which subsequently protect neuronal cells and facilitate poststroke brain recovery. While the PLC*γ* pathway has been suggested to play a role in neuroplasticity and in cell death, the PI3K/Akt and MAPK/ERK pathways mainly protect neurons from excitotoxicity and cell death, chiefly apoptosis. The PI3K/Akt signaling is also reported to support neuronal survival under oxygen/nutrient deprivation and to contribute to neurogenesis during rehabilitation.

The use of BDNF as a direct therapeutic agent for the treatment of stroke has been extensively investigated in rodent models (Figure 4). In the rat MCAO model of ischemia, pretreatment with intraventricular or intravenous BDNF drastically reduced the infarct size and the extent of neuronal cell death. It also stimulated neurogenesis, promoted sensorimotor recovery, and induced neural plasticity [62, 80, 89–91]. Grafting of BDNF-overexpressing fibroblast cells into the medial part of the somatosensory cortex lowered the number of DNA breakages and upregulated the level of full-length TrkB in the penumbra [90]. Conversely, blockade of BDNF expression by antisense oligonucleotides largely abolished the postischemia recovery of motor function and skilled reaching [79].

At the cellular level, neurogenesis and migration have been commonly used as parameters to evaluate the therapeutic effects of BDNF on stroke recovery. In a photothrombotic ischemia model, Schäbitz et al. found that intravenous injection of BDNF significantly increased the number of doublecortin-positive cells in the dentate gyrus (DG) of the hippocampus and promoted the migration of neural progenitor cells (NPCs) towards the striatum [62]. In another study using the mouse MCAO model, vasculature-dependent generation and migration of neuronal precursor cells were observed from the SVZ to the ischemic striatum, which benefitted from the sustained expression of BDNF by the endothelial cells. Also, although astrocytes did not actively express BDNF themselves, they seemed to trap a large amount of BDNF which was also able to contribute to poststroke migration of neuronal precursor cells [92].

In the exploration of immuno-/cell therapies for stroke, once again, BDNF seems to be a clear target. Intravenous transplantation of an immortal human microglial cell line into rat brains 48 hours following MCAO resulted in the upregulation of several neurotrophic factors, including BDNF, and anti-inflammatory cytokines, which helped with the functional recovery from ischemia [93]. Hydroxysafflor yellow A (HSYA) is a natural product and the active ingredient found in the Chinese herbal medicine Flos Carthami Tinctorii, which was recently shown to exert neuroprotective effects in cerebral ischemia by enhancing BDNF expression in microglia [94, 95]. In another study, intranasal delivery of BDNF was shown to increase the number of activated and phagocytotic microglia, as well as the level of cytokines and transcription factors, in order to protect brain tissue from ischemic insult and reduce neuronal injury (Figure 4) [96].

## 7. Improvement of BDNF Delivery and the Development of New TrkB Agonists

Despite all the advantages of using BDNF to fix neurological impairments, there are drawbacks to this biomacromolecule that have to be taken into consideration when viewing it as a potential product from the drug development pipeline.

First of all, although it binds less than proBDNF, BDNF still exhibits low affinity to p75^NTR^, a proapoptotic receptor that can elicit the neuronal cell death process [30]. Second, there is the matter of specificity. TrkB expresses universally in different organs of the human body and has other important physiological roles, such as the regulation of metabolism and energy homeostasis [97, 98]. Specifically, TrkB is also a therapeutic target for obesity, possibly due to its involvement in the leptin-melanocortin pathway that controls appetite. Therefore, the delivery of BDNF needs to be more specific to reduce the side effect of weight loss. Third, BDNF has a low transport rate across the intact BBB and a short serum half-life (less than 10 min), which together indicate a narrow treatment window that demands a large amount of BDNF to maintain an effective therapeutic dose. Fourth, intracranially administrated BDNF cannot diffuse into the parenchyma of targeted tissues given its tendency to be physically “sticky” (pI ≈ 10) [99]. Finally, manufacturing of BDNF remains largely limited by current technology, and consequently, the cost is correspondingly high.

To cope with these drawbacks and maximize the therapeutic efficacy of the BDNF-TrkB pathway in stroke treatment, improved BDNF delivery methods and alternative TrkB agonists have been developed in recent years. We will summarize the progress as follows (Figure 4).

### 7.1. Improvement of BDNF Delivery

In 1999, Wu and Pardridge first improved the plasma pharmacokinetics of BDNF by incorporating polyethylene glycol (PEG) moieties at the surface of its carboxyl residues. This modified BDNF was then linked to OX26mAb to facilitate capillary endothelial transferrin receptor-mediated transport through the BBB. Peripheral administration of PEG-BDNF-OX26mAb, but not BDNF alone, was able to restore the neuronal cell density in the CA1 region and significantly reduce the infarct size after transient ischemic insult in rats [100, 101].

Genetic modification may be another fast and effective method to achieve long-term and specific delivery of BDNF. For instance, when fused to a rabies virus glycoprotein-derived peptide, BDNF was able to enter nerve cells *via* intravenous administration and exhibited neuroprotective effects in a mouse model of stroke [102]. Fibrin is a protein abundantly found in hematoma surrounding the damage sites after intracerebral hemorrhage. BDNF was therefore fused with a fibrin-binding domain (FBD) to make FBD-BDNF, which could be specifically concentrated and retained at the lesion sites for a longer time than BDNF alone [103]. Similarly, fusing BDNF with a collagen-binding domain successfully retained BDNF for a longer time from degradation and promoted neurogenesis in the SVZ in an intracerebral hemorrhage model [104].

Utilizing novel drug-delivery methods, BDNF can also penetrate the BBB with higher efficacy. Harris et al. performed intravenous administration of a nanoparticle polyion complex formulation of BDNF which demonstrated better brain uptake, significantly reduced tissue loss, and increased expression of myelin basic protein [105]. With an ultrasound and microbubble system, Rodríguez-Frutos et al. delivered BDNF to the brain with higher efficiency and achieved better poststroke recovery of injured white matter, as measured by increased oligodendrocyte number and remyelination markers [106].

### 7.2. Development of New TrkB Agonists

Another solution to circumvent the disadvantages of using BDNF directly as a medication is to develop new TrkB agonists. One of the most extensively studied compounds that imitates the function of BDNF is 7,8-dihydroxyflavone (7,8-DHF), a small molecule TrkB agonist that protects neurons from kainic acid-induced toxicity and experimental ischemia [107]. 7,8-DHF efficiently induces TrkB autophosphorylation and downstream activation of various signaling pathways, including the Akt and ERK pathways. Pretreatment with 7,8-DHF *in vitro* and *in vivo* reduced the death of immature neurons in the hippocampus in a controlled cortical impact (CCI) model [108]. Postinjury treatment with 7,8-DHF within 10 min was also effective for reducing brain edema and cell death *via* Akt activation [11]. In another related study using the fluid percussion injury (FPI) model, treatment with 7,8-DHF successfully restored TrkB signaling and memory function in the Barnes maze test [109]. 7,8-DHF also exhibited beneficial effects in the treatment of neurodegenerative diseases, including Alzheimer's disease (AD), Parkinson's disease (PD), Huntington's disease (HD), and amyotrophic lateral sclerosis (ALS) [110–114]. Recently, a derivative of 7,8-DHF, namely, BrAD-R13, was approved by the FDA for use in clinical trials for the treatment of mild to moderate AD.

A pharmacophore proven to be critical in activating TrkB signaling is the BDNF mimetic LM22A4, which activates the Akt and ERK signaling pathways comparably to BDNF. Massa et al. performed intranasal administration of LM22A4, in *in vitro* neurodegenerative disease models to promote cell survival, and in a rat parietal CCI model to reduce neuronal cell death [115]. Based on the sequence of BDNF loop 4 and loop 1 *β*-turn, Gudasheva and colleagues have recently created two dimeric dipeptides, which mimic full-length BDNF. They exhibited varied abilities to activate the Akt and ERK pathways and exhibited therapeutic effects in the rat MCAO model [116].

As the strategy of immunotherapy has prevailed in recent years, several specific TrkB agonistic antibodies have been developed which exhibited promising preclinical effects in the treatment of acute and chronic neurological diseases. Recent studies suggest that TrkB agonistic antibodies bind to the receptor with high specificity and affinity to activate its downstream signaling cascades; thus, they exhibit protective effects in ischemic stroke [10, 117], HD [118], and glaucoma [119] and regulate energy metabolism [120]. Using a human short-chain variable fragment antibody library, Merkouris et al. screened new TrkB agonistic antibodies and characterized their function at the cellular level. These antibodies induced TrkB phosphorylation, downstream signaling pathway activation, and gene expression to comparable levels as BDNF [121]. Together, these studies made great strides toward BDNF-based therapeutics for neurological diseases.

## 8. To Avoid Preclinical Research Pitfalls

While novel therapeutic targets have emerged from preclinical studies, none has yet been proven to be as effective in clinical trials. The Stroke Therapy Academic Industry Roundtable has reported recommendations on preclinical drug discovery for ischemia that may shed light on the development of therapeutic plans, which would lower the risk of wasted effort on, and money in, failing clinical trials [122].

First of all, a good preclinical study generally starts with the right animal models. Rodents are usually considered at the first place, as the scientific community has a wealth of experience in using them to model brain diseases. Second, the candidate drugs should be monitored closely based on their mechanisms of actions, functional and pathological outcomes, and physiological properties. For the neuroprotective drugs that exhibit efficacy at the time of damage, it is important to plot dose-response and administration time-response curves in order to learn the effective range of concentrations and functional time windows in the acute phase of disease onset; while for the neurorestorative drugs, long-term monitoring of functional outcomes is more important. Last but not the least, to avoid bias and increase the reproducibility of the data, the experiments should be carried out in at least two laboratories independently in a blinded, randomized fashion with both rodents and upper-level species, preferably with nonhuman primates [122].

## 9. Conclusions

To date, the role of the BDNF-TrkB pathway in mediating functional rehabilitation and recovery from stroke has been extensively investigated. A great number of studies suggest that BDNF exerts favorable effects in poststroke recovery due to its attenuation of cell death, promotion of neurogenesis/-migration, and remyelination of axons, as well as its ability to interfere with neuroinflammatory factors and cells. Nevertheless, more detailed mechanisms of the BDNF-TrkB pathway's effect on stroke recovery, or that of other brain insults, remain elusive and will require further in-depth investigation. Moreover, due to the unfavorable physical and biochemical properties of BDNF as a treatment agent, the prospect of its clinical use has been greatly hampered. Therefore, the use of new technologies to manufacture BDNF, innovative strategies to control its targeted delivery, and production of BDNF mimetics in comprehensive preclinical studies may pave the way for future clinical application of the BDNF-TrkB signaling pathway in stroke treatment.
